# Metabolic Variation among Fruits of Different Chili Cultivars (*Capsicum* spp.) Using HPLC/MS

**DOI:** 10.3390/plants11010101

**Published:** 2021-12-29

**Authors:** Tilen Zamljen, Aljaž Medič, Robert Veberič, Metka Hudina, Jerneja Jakopič, Ana Slatnar

**Affiliations:** Department of Agronomy, Biotechnical Faculty, University of Ljubljana, SI-1000 Ljubljana, Slovenia; aljaz.medic@bf.uni-lj.si (A.M.); robert.veberic@bf.uni-lj.si (R.V.); metka.hudina@bf.uni-lj.si (M.H.); jerneja.jakopic@bf.uni-lj.si (J.J.); ana.slatnar@bf.uni-lj.si (A.S.)

**Keywords:** capsaicinoids, metabolomics, organic acids, pungent, sugars

## Abstract

Chilies are widely cultivated for their rich metabolic content, especially capsaicinoids. In our study, we determined individual sugars, organic acids, capsaicinoids, and total phenolic content in pericarp, placenta, and seeds of *Capsicum annuum* L., *Capsicum chinense* Jacq. and *Capsicum baccatum* L. by HPLC/MS. Dry weight varied in the cultivar ‘Cayenne’, with the first fruit having the lowest dry weight, with 4.14 g. The total sugar content and organic acid content did not vary among the fruits of all three cultivars. The cultivar ‘Cayenne’ showed differences in total phenolic and capsaicinoid content between fruits in the placenta, with the first fruit having the highest content of total phenolics (27.85 g GAE/kg DW) and total capsaicinoids (16.15 g/kg DW). Of the three cultivars studied, the cultivar ‘Habanero Orange’ showed the least variability among fruits in terms of metabolites. The content of dihydrocapsaicin, nordihydrocapsaicin, homocapsaicin, and homodihydrocapsaicin in the seeds of the second fruit was higher than that of the first fruit of the cultivar ‘Bishop Crown’. The results of our study provided significant insight into the metabolomics of individual fruits of the same chili plant. We have thus increased our understanding of how certain metabolites are distributed between fruits at different levels of the same plant and different parts of the fruit. This could be further investigated when chilies are exposed to different environmental stresses.

## 1. Introduction

Chilies are widely cultivated through many parts of the world, especially in South America and Asia [1]. In many cultures, they are considered one of the most important foods for consumption. There are six species of chilies that are widely cultivated, of which *Capsicum annuum* L. and *Capsicum chinense* Jacq. are the two most cultivated, followed by *Capsicum baccatum* L. and *Capsicum frutescens* L. [2,3]. 

A chili plant reaches between 0.5 and 1.5 m in size when mature and has dichotomous growth [1]. The basic principle of dichotomous growth or dichotomous branching is that a plant shoot always splits into two new shoots [4]. This type of growth is typical of pungent chilies or sweet peppers. On each section where the shoots separate, flowers are formed. In *C. annuum* and *C. baccatum*, one flower (one fruit) is formed at each separation and in *C. chinense* two to four flowers are formed (resulting in two to four fruits) at one dichotomous branch (node) [5]. Depending on the cultivar and species, chili fruits can grow erect or pendent [1]. Chili fruits of cultivars that produce only one fruit per node are generally larger than those that produce two to four fruits on one node [1].

The flowering of chili fruits is acropetal, which means that the flowers develop, mature or open at the first dichotomous branching of the plant, with further sections following in sequence. The fruit are therefore also set in sequential order. The first fruit forms at the first node, the second forms at the second node, and so on [4].

Chilies are a great source of vitamin C (ascorbic acid) and capsaicinoids (capsaicin, dihydrocapsaicin, nordihydrocapsaicin, homocapsaicin, and homodihydrocapsaicin) [6]. There are many ways of influencing the synthesis of primary and secondary metabolites in chilies. These include genetics (new varieties, breeding, tissue cultures etc.) or different agricultural methods, such as fertilization, irrigation, light intensity, etc. Agricultural methods can also be used as controlled stress on plants to promote the biosynthesis of these metabolites [7,8,9].

In our study we tested whether chilies grown under normal conditions produce fruits of different quality on the same plant. The competition for photosynthetic metabolites increases with the number of fruits per plant [10]. The first fruit usually has the most resources for growth and development, due to the lack of other fruits, so the first fruit may contain more primary and secondary metabolites. This raises many questions regarding chili production: (i) does the metabolic constitution change between different fruits of the same plant, (ii) are there enough sugars to supply all the synthesis pathways in each fruit of the plant and (iii) are there differences among chili species when it comes to fruit variability? Since these questions were previously insufficiently addressed in other studies, our study provides answers to these crucial questions. Additionally, the study’s goal was to investigate whether picking fruit from certain levels on the plant can influence their quality in terms of metabolites, which could consequently lead to a targeted harvest as fruit quality is important for consumers as well as for the pharmaceutical industry.

## 2. Materials and Methods

A pot experiment was conducted at the Biotechnical Faculty, Ljubljana (46°3′25″ N; 14°30′20″ W) in a plastic greenhouse. Chili seeds were sown in February. The seeds of all three cultivars were purchased at Austrosaat. In May, the seedlings were planted in plastic pots (φ 30 cm), filled with peat substrate (Neuhaus N3), and placed in a plastic greenhouse. Each pot was watered and fertilized through a drop irrigation system to provide equal amounts of water and fertilizer to each plant. The average temperature during the experiment was 23.5 °C and relative humidity 79.1%. The experiment ended on 15 October 2019 when the last fruits were picked.

**Figure 1:**
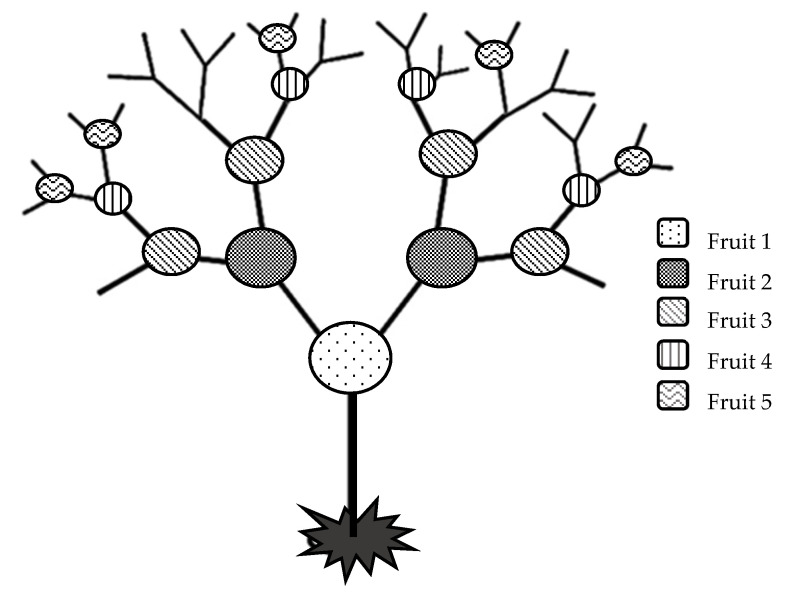
Level of fruit picking position on plant.

The fruits were picked from three different species (*C. annuum*, *C. chinense,* and *C. baccatum*), when the cultivar-specific color was reached. In *C. annuum* ‘Cayenne’ and *C. baccatum* ‘Bishop Crown’, the color at harvest was red and in *C. chinense* ‘Habanero Orange’ orange as reported by Zamljen, et al. [11]. For each cultivar, fruits of six plants from the first, second, third, fourth, and fifth levels were collected (Figure 1), except for *C. chinense* ‘Habanero Orange’ where only four levels of fruit of could be picked. Each individual fruit was first weighed and then the three fruit parts (pericarp, placenta, and seeds) were separated with a scalpel. All fruit parts were then lyophilized (freeze-dried) individually and stored at −20 °C until sugar, organic acids, total phenols, and capsaicinoids were extracted.

### 2.1. Extraction of Sugars, Organic Acids and Ascorbic Acid

Lyophilized powder (0.05 g for pericarp and placenta) was extracted with 2 mL of bidistilled water for the analysis of sugars and organic acids. The extraction of ascorbic acid was carried out with 0.05 g of dried powdered sample, extracted with 4 mL of 2% metaphosphoric acid. The samples were then shaken (Heidolph, Unimax 1010) for 30 min and, after shaking, transferred to a (4 °C) centrifuge (Eppendorf Centrifuge 5810 R) in which the samples were centrifuged at 10,000× *g* for 8 min. The supernatant was then filtered through a 0.25 µm cellulose filter (Chromafil A-25/25; Macherey-Nagel, Dueren, Germany) and placed in a freezer at −20 °C, until further analysis. 

### 2.2. Analysis of Sugars, Organic Acids and Ascorbic Acid

The analysis was performed on the Thermo Finnigan Surveyor HPLC system equipped with an IR (infrared) detector for sugar analysis and UV (ultraviolet/visible light) detector for organic acids. (Thermo Scientific, San Jose, CA, USA). For sugars, a Rezex RCM monosaccharide (30 × 0.78 cm; Thermo Scientific, San Jose, CA, USA) column and for organic acids and ascorbic acid Rezex ROA an organic acid column (30 × 0.78 cm; Phenomenex, Torrance, CA, USA) were used. Both columns were heated to 65 °C, except for ascorbic acid where the column was heated to 25 °C. The adjustment of the HPLC system was based on Zamljen, et al. [12]. For the analysis of sugars an isocratic method was used with the mobile phase consisting of bidistilled water. For the analysis of organic acids and ascorbic acid an isocratic method was used with the mobile phase consisting of 4 mM H_2_SO_4_ in bidistilled water. The results were expressed in g/kg dry weight (DW) for sugars, organic acids and ascorbic acid. The chromatographic data for sugars are presented in Figure S1 and chromatographic data for organic acids are presented in Figure S2A. The chromatographic data for ascorbic acid are presented in Figure S2B.

### 2.3. Extraction of Total Phenolics and Capsaicinoids

The extraction method was the same for total phenolics and capsaicinoids, using 0.05 g of the dry pericarp, 0.05 g of dry seed sample, and 0.02 g of the dry placenta sample. Samples (pericarp, placenta, and seeds) were extracted with 4 mL 80% methanol in water. After the samples were placed in an ultrasonic bath (Sonis 4 ultrasonic bath; Iskra pio, Sentjernej, Slovenia) they were cooled to 0 °C for 60 min. After, the samples were placed in a cooled (4 °C) centrifuge (Eppendorf Centrifuge 5810 R) set to 8000× *g* for 6 min. Supernatant was then filtered through a 0.25 µm polyamide filter (Chromafil AO -45/25, Macherey-Nagel, Dueren, Germany) into a vial. 

### 2.4. Analysis of Total Phenolic

The Folin-Ciocalteu (FC) phenol reagent method [13] was used for the determination of total phenolic content (TPC) in chilies. A total 100 μL of the sample, 500 μL of FC, 1.5 mL 20% Na_2_CO_3_, and 7.9 mL of bidistilled water were mixed and placed in an oven preheated to 40 °C. The absorbance at 765 nm was determined with a Lambda Bio 20 UV/VIS spectrophotometer (Perkin Elmer, Waltham, MA, USA). The total phenol content was expressed as gallic acid equivalents (GAE) in g/kg dry weight (DW).

### 2.5. Analysis of Capsaicinoids

The same extract was used for the analysis of capsaicinoids, as for TPC. For quantification of capsaicin, dihydrocapsaicin, nordihydrocapsaicin, homocapsaicin, and homodihydrocapsaicin, a UHPLC Thermo Scientific Dionex UltiMate 3000 HPLC (Thermo Scientific) system coupled with a TSQ Quantum Access Max quadrupole mass spectrometer (Thermo Scientific, Waltham, MA, USA) was used. A Gemini C18 (Gemini; 150 × 4.60 mm, 3 u; Phenomenex, Torrance, CA, USA) column operating at 25 °C was used to separate the individual capsaicinoids. The settings of the HPLC/MS system were H-ESI source in positive ion mode at vaporizer temperature 300 °C, spray voltage 4.5 kV, sheath gas 50 arbitrary units (au), auxiliary gas 20 arbitrary units, ion transfer capillary temperature 320 °C. The sampled mass spectra range was from *m*/*z* 90 to 700. Argon was used as the collision gas. The SRM mode (selected-reaction monitoring mode) was used for data acquisition. Other parameters of the HPLC/MS were based on Zamljen, et al. [14] and Medic, et al. [15]. Homocapsaicin and homodihydrocapsaicin were expressed as capsaicin and dihydrocapsaicin equivalents, respectively. The chromatographic data for capsaicin, dihydrocapsaicin, and homodihydrocapsaicin are presented in Figures S1–S3. All five capsaicinoids were expressed in g/kg DW.

### 2.6. Chemicals

The following standards were used: For sugars sucrose, glucose and fructose (Sigma–Aldrich Chemie GmbH, Steinheim, Germany); for organic acids oxalic acid, citric acid, malic acid, succinic acid, quinic acid, ascorbic acid and fumaric acid (Sigma Aldrich Chemie GmbH, Steinheim, Germany); for capsaicnoids capsaicin, dihydrocapsaicin and nordihydrocapsaicin (Sigma–Aldrich Chemie GmbH, Steinheim, Germany).

### 2.7. Statistical Analysis

The statistical analyses were performed within the R environment [16]. For *C. annuum* ‘Cayenne’ variety, 13 pepper fruits were picked. Fruit weight and the concentrations of 16 individual metabolites (capsaicinoids, sugars, organic acids, and phenolics) were analyzed, and the total concentration was calculated as the sum of the corresponding concentrations. For each variable of interest, statistical analysis was performed on the logarithmic scale. Two factors were studied: fruit position (Fruit 1, Fruit 2, …, Fruit 5) and fruit part (placenta, pericarp, seed). To account for the fact that values on individual fruits were correlated, mixed model methodology (R package nlme) was used. Comparisons with the base position L1 were evaluated (R package multcomp). 

**Figure 2:**
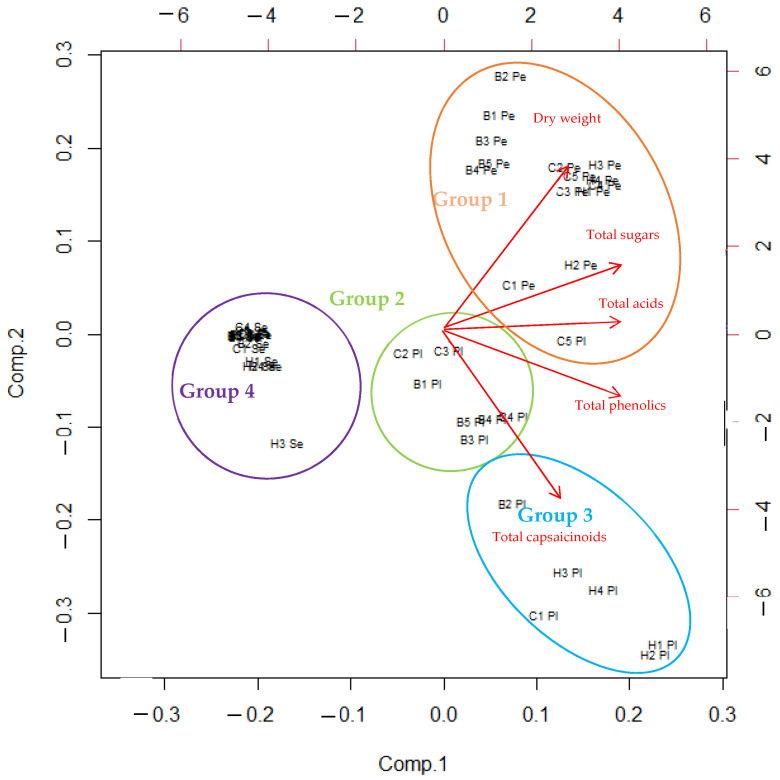
Biplot corresponding to PCA for all samples. **Pe** = pericarp; **Pl** = placenta; **Se** = seed; **C** = ‘Cayenne’; **H** = ‘Habanero Orange’; **B** = ‘Bishop Crown’; cultivar labels were coded 1 to 5 according to picking position within the plant (1, innermost; 5, outermost); **Group 1 to 4** = Grouping of samples based on multivariate analysis.

A principal component analysis (PCA) was performed for all data (Figure 2). The first and second components of the full-data PCA model accounted respectively for 62.3 and 26.3 % of total variance. A bi plot was made to visualize the results of the PCA (Figure 2).

## 3. Results 

### 3.1. Multivariate Analysis of All Data 

PCA of all samples and metabolites was performed to provide a comprehensive picture of our study (Figure 2). All the three cultivars, all fruit levels, and all the three fruit parts were considered. Four groups were formed. The first consisted of all pericarp samples (Pe) of all three cultivars one placenta (Pl) sample of the fifth ‘Cayenne’ fruit. This group had high dry weight, total sugar content, and total acidity. The second group consisted of all seed samples (Se), which had low metabolite contents. The third group consisted of the placenta of the second, third, and fourth fruits of ‘Cayenne’ and the first, third, fourth, and fifth fruits of ‘Bishop Crown’ and all contained average dry weight, high primary metabolite content, and high phenolic and capsaicinoid content. The fourth group consisted of fruits that had very high capsaicinoid and phenolic contents. All four fruits of the variety ‘Habanero Orange’, the first fruit of the variety ‘Cayenne’, and the second fruit of the variety ‘Bishop Crown’ belonged to this group.

### 3.2. Fruit Weight

The average weight of the whole fruit and of each individual fruit part is shown in Table 1. In the *C. chinense* ‘Habanero Orange’ cultivar the fruits on the second level had lower dry weight in pericarp and in the whole fruit, compared to the fruits on the first level. On the other hand, in the *C. baccatum* ‘Bishop Crown’ the fruits on the second level had higher dry weight compared to the fruits on the first level. The fruits on the fifth level had lower dry weight compared to the fruits on the first level. Statistically significant differences were found in the whole fruit of *C. annuum* ‘Cayenne’ and all three fruit parts. Fruits on the second level had the highest pericarp weight (11.75 g) and fruits on the first level had the lowest pericarp weight. The fruits on the first level in ‘Cayenne’ also had the lowest weight of placental tissue (0.26 g) and seeds (0.47 g). When observing whole fruit data, the second fruit of the ‘Cayenne’ (13.71 g) had the highest fruit weight. The first fruit of the ‘Cayenne’ on the other hand had the lowest fruit weight (4.14 g).

Based on PCA of all data (Figure 2), we found that the pericarp group consisted of fruit parts with high dry weight. High dry weight was present in all pericarps of all three cultivars and in the fifth fruit of ‘Cayenne’ placenta. Medium dry weight was present in the other fruits of ‘Cayenne’ and all fruits of ‘Habanero Orange’ and ‘Bishop Crown’. Low dry weight was present in all seed samples of all three cultivars.

### 3.3. Sugars

No statistical differences were observed in the total sugar content of all three cultivars (Table 2). In the ‘Cayenne’ cultivar statistical differences were observed in individual sugar contents. There were statistically significant differences in the placenta for all three sugars analyzed and in the pericarp for both glucose and fructose. The fruits on the fifth level in ‘Cayenne’ also had the highest content of all three analyzed sugars in both fruit parts compared to the fruits on the first level. In the placenta, the lowest content of sucrose, glucose, and fructose was found in the fruits on the first level. There were no statistically significant differences in the total sugar content and individual sugar content in the pericarp and placenta of each fruit in ‘Habanero Orange’. In ‘Bishop Crown’, no statistically significant differences were found in the pericarp and placenta total sugar content. In the placental tissue, only statistical differences in sucrose were found, with the fruits on the fourth level showing the highest content, 120.77 g/kg DW, which is an 8-time increase compared to the fruits on the first level. The fruits on the second level of ‘Bishop Crown’ had 16.12 g/kg DW higher glucose contents in the placenta compared to the fruits on the first level. No statistical differences were found in fructose content in the placenta. In the pericarp, however, no statistical differences were found for both glucose and fructose. In all three cultivars, fructose was the most common sugar, followed by glucose and sucrose. No statistically significant differences were observed in the total sugar contents in ‘Bishop Crown’ and ‘Habanero Orange’. The variation in these two cultivars was the least among the different fruits.

PCA of all data (Figure 2) showed that the pericarp group, which had a high dry weight, also contained a high total sugar content. It consisted of all pericarp samples and the fifth fruit ‘Cayenne’ placenta sample. The other three groups (seed group, first and second placenta groups) had medium total sugar contents as in the case of the first and second placenta groups and low sugar contents in the seed group which contained all the seed samples.

### 3.4. Organic Acids

The individual and total organic acids per sample are shown in Table 3. No statistically significant differences were found for ‘Cayenne’, ‘Habanero Orange’, and ‘Bishop Crown’ with regard to the total organic acid content in pericarp and placenta.

Statistically significant differences were observed in certain individual organic acids. The fruits on the fourth level of ‘Cayenne’ had the highest contents of malic acid, quinic acid, and oxalic acid in pericarp and the highest contents of quinic acid in placenta (Table 3) compared to the fruits on the first level. The fruits in the third and fourth level of ‘Habanero Orange’ had 9.22 g/kg DW and 11.30 g/kg DW less malic acid in the pericarp, when compared to the fruits on the first level respectively. The fruits on the first level of the ‘Habanero Orange’ cultivar had the highest contents of citric and quinic acid in placenta. In ‘Bishop Crown’ the fruits on the third level placenta had 25.99 g/kg DW less citric acid content compared to the fruits on the first level.

Ascorbic acid contents varied only in the ‘Cayenne’ cultivar. The fruits on the third level had 4.71 g/kg DW less ascorbic acid compared to the fruits on the first level in the pericarp. In the placenta the lowest content of ascorbic acid was present in the fruits on the third level (Table 3), with 0.70 g/kg DW.

PCA of all data (Figure 2) showed that total acid contents were high in the pericarp group, with particularly high contents in the fifth fruit placenta of ‘Cayenne’ and the second fruit pericarp of ‘Habanero Orange’. Most samples had average total acid contents and seed samples had low organic acid contents.

### 3.5. Total Phenolic Contents and Capsaicnoid Contents 

#### 3.5.1. Total Phenolic Contents

Total phenolic content in different fruit parts and whole fruit is shown with Table 4. The placenta generally shows higher phenolic contents and is the main reason for variations in the whole fruit. The fruits on the first level of ‘Cayenne’ had the highest total phenolic content in whole fruit (10.76 g/kg DW). All other fruits of ‘Cayenne’ had a lower total phenolic content. In ‘Habanero Orange’, there were no significant differences in total phenolic content in all three fruit parts and also in whole fruit. The fruits on the second level of ‘Bishop Crown’ had the highest content of total phenolics in placenta with 19.01 g/kg DW (Table 4) or compared to the fruits on the first level. Total phenolic content was highest in the placenta of all studied cultivars, followed by pericarp and seeds. Statistical differences were found only in the placenta of ‘Cayenne’ and ‘Bishop Crown’, not in pericarp and seeds. 

PCA of all data (Figure 2) showed that none of the samples stood out in terms of total phenolics. Most of the pericarp groups and the first placenta group had average total phenolic content. The second placenta group, formed by placenta samples from the second ‘Bishop Crown’ fruit, the first ‘Cayenne’ group, and all four ‘Habanero Orange’ fruits, contained high phenolic contents. The seed samples contained low total phenolic contents.

#### 3.5.2. Capsaicinoids

Noticeable variations were present in capsaicinoids content among different fruits in the ‘Cayenne’ cultivar (Table 5). ‘Cayenne’ fruits on the second and third level had lower capsaicin, dihydrocapsaicin, nordihydrocapsaicin, and homocapsaicin compared to the fruits on the first level. The fruits on the first level had the highest content of capsaicinoids in placenta (16.15 g/kg DW) with approximate 4.6-fold increase compared to the fruits on the second and third level. No differences could be observed in the ‘Habanero Orange’ cultivar individual and total capsaicinoid content. ‘Bishop Crown’ fruits on the second level had the highest content of dihydrocapsaicin, nordihydrocapsaicin homocapsaicin, and homodihydrocapsaicin in seeds. In all three cultivars, the composition of capsaicinoids was similar among fruits picked from different levels. In all fruits, capsaicin and dihydrocapsaicin represented the majority of all capsaicinoids, followed by nordihydrocapsaicin, homocapsaicin, and homodihydrocapsaicin in a less extent. In all three species the placenta had the highest content of capsaicinoids followed by pericarp and seeds. 

By PCA of the entire data (Figure 2), we found that the second placenta group, which consisted of placenta samples from the second ‘Bishop Crown’ fruit, the first ‘Cayenne’ group, and all four ‘Habanero Orange’ fruits, had very high total capsaicinoid contents. The pericarp group and the first placenta group all contained samples that had average capsaicinoid contents and the seed group all contained seed samples that had low capsaicinoid contents.

## 4. Discussion

We investigated the possible effects of fruit positioning on the quality of metabolites in the fruits of three different chili cultivars. The results show that the variation is cultivar specific. In the cultivar ‘Cayenne’, the fruits at the first level usually have the highest weight, probably due to the greater availability of photosynthetic metabolites [17]. In our study, this was not the case as the fruits on the first level did not have the highest weight. The pericarp had the highest weight compared to the other two fruit parts, which is consistent with the result previously reported by Krstić, et al. [18]. One possible reason for the lower fruit weight at the first stage in ‘Cayenne’ is the rapid plant growth after planting. *Capsicum* plants usually form the first flower bud or even the first fruit after transplanting in the field. They invest most of all metabolic products in the vegetative growth of the plant, so fruits may be smaller than later fruits when vegetative growth slows down [1]. 

As previously reported by Pandhair and Sharma [19] and Aldana-Iuit, et al. [20], capsaicinoid content was highest in placenta and is consistent with our results. Capsaicin was most abundant in all three parts of the fruit followed by dihydrocapsaicin, nordihydrocapsaicin, homodihydrocapsaicin, and homocapsaicin. The same results were previously reported by Gavilán Guillen, et al. [21], Loizzo, et al. [22] and Loizzo, et al. [23]. In all cultivars, the fruits had the highest content of capsaicinoids at the first or second stage. Similar results were also reported by Zewdie and Bosland [4], where fruits at the second level (the first fruit was not analyzed) had the highest capsaicinoid content, followed by other fruits from higher levels. 

Differences between fruits in primary and secondary metabolites may be related to several factors, including environment or genotype [24]. Fruits higher in the canopy are exposed to more light. Iwai, et al. [25] reported that light intensity can affect the level of metabolites in chilies. Fruits located further down the plant are covered by leaves and have much less sunlight available [26]. Higher sunlight exposure results in larger fruits and higher yields [27]. Metabolites such as capsaicinoids or phenols decrease with fruit maturity and ripeness [28]. Several enzymes, such as peroxidases, cause this decline. Peroxidase activity increases as the fruit ripens. The longer we wait to pick the fruit, the less metabolites such as capsaicinoids remain [29]. Estrada, et al. [30] reported similar results, where the first stage fruits did not have the highest content of phenolics and capsaicinoids. In cherry tomatoes, it has been reported that the sugar and phenolic content of fruits varies, although this variation is cultivation specific [31], similar is reported by Loizzo, Pugliese, Bonesi, Menichini, and Tundis [23] in chilies total phenolics contents. Higher fruits have a greater amount of nutrients available than the first fruits because the photosynthetic rate is higher and so is transpiration, resulting in better nutrient uptake (larger leaf area) [32]. With the higher photosynthetic rate, the sugar content also increases, which would explain the higher glucose and fructose content of the cultivar ‘Cayenne’. In the cultivar ‘Habanero Orange’, we also observed that there were fewer differences between fruits in sugars, organic acids, phenols, and capsaicinoids. The reason for this occurrence is genetic. The plant may have a better transport system for metabolites between different parts of the plant, especially sugars, which are the main synthetic substrate for other metabolites [31]. It may be more photosynthetically active and receive sufficient sugars for normal fruit development from the beginning, regardless of the height of the plant [10]. The organic acid results indicate that there is less variation among fruits than other metabolites. In apples, fruit position in the canopy did not affect inter-fruit variation in organic acids such as malic acid, and the small differences that occurred in specific fruit parts or cultivars were cultivar dependent rather than a general rule [33], which is consistent with our results in chilies and was proved by PCA (Figure 2).

Chili plants distribute metabolites differently among fruits depending on the growth conditions of the plant and the stage of fruit development and maturity [34]. The genotype of the cultivar itself and its influence on fruit variability is also an important factor. Factors such as irrigation, light intensity, and fertilization have a much greater influence on fruit quality than picking only the first three or four fruits with minimal differences between them. It is also questionable whether it is ecologically justifiable for farmers to pick only certain fruits of the whole plant.

## 5. Conclusions 

This study gave a significant insight into the metabolic picture of each fruit on the same plant. As far as we know, few studies have been conducted to investigate the effects of fruit positioning in the canopy of chili plants on their metabolic content and variation. We thus extended our understanding of how certain metabolites are distributed between fruits at different levels of the same plant and different parts of the fruit. Our study showed large variations in the *C. annuum* species in terms of the metabolite variations among fruits. With respect to a health view on overall fruit quality, the influence of fruit positioning is not significant enough in the other two species. Fruit positioning in chilies could be an interesting study topic in the future, whereby chili plants could be exposed to various environmental stresses such as drought and light stress to determine if individual fruit react differently to these stressors on the same plant. 

## Figures and Tables

**Table 1:** Average dry weight of individual fruit parts and whole fruit (g; mean ± SE).

Treatment	Dry Weight	Level 1	Level 2	Level 3	Level 4	Level 5
		Mean ± SE	Mean ± SE	Mean ± SE	Mean ± SE	Mean ± SE
*C. annuum* ‘Cayenne’	Pericarp	3.72 ± 0.21	11.75 ± 1.71 ***	9.33 ± 0.36 *	9.51 ± 0.67 *	8.92 ± 0.88 *
	Placenta	0.26 ± 0.01	0.73 ± 0.16 *	0.72 ± 0.06 **	0.74 ± 0.01 **	0.86 ± 0.12 ***
	Seed	0.47 ± 0.01	1.34 ± 0.12 *	1.36 ± 0.12 *	1.44 ± 0.06 **	1.22 ± 0.16
	**Whole fruit**	**4.14 ± 0.31**	13.71 ± 1.23 ***	11.35 ± 0.93 **	11.62 ± 0.86 **	10.95 ± 1.01 **
*C. chinense* ‘Habanero Orange’	Pericarp	12.61 ± 1.11	9.25 ± 0.87 **	14.71 ± 1.34	14.16 ± 1.11	
	Placenta	1.82 ± 0.63	1.56 ± 0.26	2.42 ± 0.13	2.17 ± 0.46	
	Seed	0.82 ± 0.16	0.47 ± 0.02	0.81 ± 0.26	0.87 ± 0.13	
	**Whole fruit**	**15.23 ± 1.32**	11.19 ± 0.77 **	**17.92 ± 1.45**	**17.08 ± 1.63**	
*C. baccatum* ‘Bishop Crown’	Pericarp	10.17 ± 1.13	12.63 ± 1.08 **	9.30 ± 0.76	8.18 ± 0.33	7.53 ± 0.79 ***
	Placenta	0.57 ± 0.07	0.72 ± 0.13	0.47 ± 0.17	0.52 ± 0.12	0.58 ± 0.06
	Seed	0.82 ± 0.07	1.08 ± 0.12	0.72 ± 0.27	0.67 ± 0.22	0.61 ± 0.07
	**Whole fruit**	**11.48 ± 0.52**	14.32 ± 1.27 ***	**10.48 ± 0.92**	9.22 ± 0.37 **	8.67 ± 0.52 ***

* symbol indicates statistical significant differences of each fruit and fruit part when comparing to the first fruit on the plant. Significance codes: *** ≤ 0.001; ** ≤ 0.01; * ≤ 0.05; ‘empty slot’ ≥ 0.05.

**Table 2:** Individual and total sugars (g/kg DW; mean ± SE) in three different chili cultivars fruits.

Species	Sugar	Fruit Part	Level 1	Level 2	Level 3	Level 4	Level 5
			Mean ± SE	Mean ± SE	Mean ± SE	Mean ± SE	Mean ± SE
*C. annuum* ‘Cayenne’	Sucrose	Pericarp	17.26 ± 1.23	19.21 ± 1.89	14.11 ± 0.82	22.53 ± 0.36	27.10 ± 1.21
		Placenta	4.22 ± 0.11	41.51 ± 3.20 ***	16.94 ± 1.85 *	17.89 ± 1.21	95.69 ± 7.61 ***
	Glucose	Pericarp	143.72 ± 3.71	134.96 ± 8.16	159.47 ± 1.76 **	166.77 ± 2.32 **	201.93 ± 4.12 ***
		Placenta	23.34 ± 1.53	43.85 ± 2.64	70.20 ± 6.77 **	79.81 ± 6.83 **	138.31 ± 3.36 ***
	Fructose	Pericarp	147.28 ± 7.37	140.43 ± 2.91	159.22 ± 3.41	162.71 ± 5.37	179.31 ± 3.47 **
		Placenta	68.67 ± 4.98	79.42 ± 7.38	150.11± 3.61 ***	155.94 ± 7.26 ***	236.35 ± 7.12 ***
	**Total sugars**	**Pericarp**	**308.12** **± 13.22**	**294.52** **± 10.86**	**332.79** **± 15.32**	**351.94** **± 14.35**	**408.30** **± 20.46**
		**Placenta**	**96.18** **± 8.34**	**164.74** **± 11.46**	**237.18** **± 16.33**	**253.59** **± 17.24**	**470.26** **± 19.68**
*C. chinense* ‘Habanero Orange’	Sucrose	Pericarp	21.81 ± 1.81	17.21 ± 1.72	19.80 ± 0.93	14.62 ± 1.31	
		Placenta	15.91 ± 0.91	89.41 ± 9.31	11.33 ± 0.28	14.29 ± 1.42	
	Glucose	Pericarp	169.43 ± 8.23	163.46 ± 4.48	171.55 ± 4.36	149.46 ± 9.16	
		Placenta	136.99 ± 6.28	102.89 ± 9.20	88.77 ± 7.12	122.91 ± 4.75	
	Fructose	Pericarp	216.35 ± 9.17	199.13 ± 2.63	203.11 ± 0.71	203.39 ± 12.52	
		Placenta	193.11 ± 3.91	155.63 ± 8.84	114.46 ± 4.37	153.33 ± 2.81	
	**Total sugars**	**Pericarp**	**407.57** **± 20.79**	**379.68** **±19.25**	**394.25** **± 19.92**	**367.30** **± 18.41**	
		**Placenta**	**345.96** **± 17.52**	**347.83** **± 18.31**	**214.37** **± 15.53**	**290.43** **± 20.42**	
*C. baccatum* ‘Bishop Crown’	Sucrose	Pericarp	19.14 ± 0.58	31.61 ± 1.22 **	32.11 ± 1.42 **	13.95 ± 1.97	30.71 ± 2.27
		Placenta	15.28 ± 2.50	48.57 ± 4.89	58.35 ± 2.07	120.77 ± 5.41 ***	60.55 ± 1.47
	Glucose	Pericarp	197.35 ± 3.62	181.48 ± 3.45	168.04 ± 1.09	153.32 ± 11.12	169.27 ± 5.82
		Placenta	69.42 ± 1.11	85.52 ± 3.65 *	64.47 ± 1.92	71.41 ± 2.77	59.21 ± 3.58
	Fructose	Pericarp	196.42 ± 11.14	170.82 ± 11.21	165.41 ± 15.91	161.38 ± 10.56	195.11 ± 9.31
		Placenta	130.70 ± 3.63	113.68 ± 2.57	166.11 ± 3.56	152.33 ± 3.52	139.06 ± 8.14
	**Total sugars**	**Pericarp**	**412.83** **± 22.41**	**383.84** **± 21.85**	**365.53** **± 25.36**	**328.59** **± 15.35**	**395.02** **± 17.94**
		**Placenta**	**215.30** **± 20.36**	**247.61** **± 18.94**	**288.84** **± 20.41**	**344.48** **± 19.09**	**258.73** **± 16.59**

* symbol indicates statistical significant differences of each fruit and fruit part when comparing to the first fruit on the plant. Significance codes: *** ≤ 0.001; ** ≤ 0.01; * ≤ 0.05; ‘empty slot’ ≥ 0.05.

**Table 3:** Individual and total organic acids content (g/kg DW, mean ± SE) in different species and two different fruit parts.

Species	Organic Acid	Fruit Part	Level 1	Level 2	Level 3	Level 4	Level 5
			Mean ± SE	Mean ± SE	Mean ± SE	Mean ± SE	Mean ± SE
*C. annuum* ‘Cayenne’	Ascorbic acid	Pericarp	18.11 ± 1.19	15.12 ± 1.50	13.40 ± 0.15 ***	15.37 ± 0.54	16.89 ± 0.78
		Placenta	4.27 ± 0.04	3.60 ± 0.03	0.70 ± 0.05 ***	0.91 ± 0.03 ***	2.81 ± 0.19
	Citric acid	Pericarp	73.84 ± 6.62	63.91 ± 3.82	75.42 ± 5.55	88.96 ± 5.30	80.06 ± 4.42
		Placenta	55.82 ± 4.96	68.99 ± 6.22	63.52 ± 2.21	60.31 ± 3.22	98.93 ± 4.32
	Malic acid	Pericarp	14.46 ± 1.72	15.02 ± 1.95	21.97 ± 2.03 **	24.74 ± 1.55 ***	14.43 ± 0.93
		Placenta	35.76 ± 2.02	27.32 ± 1.84	32.04 ± 3.15	48.94 ± 3.74	45.96 ± 1.70
	Quinic acid	Pericarp	11.32 ± 1.06	10.77 ± 0.92	15.32 ± 1.26 *	18.37 ± 1.42 *	12.72 ± 0.99
		Placenta	12.60 ± 0.56	9.84 ± 0.42	12.36 ± 1.02	14.82 ± 1.36	14.42 ± 1.66
	Succinic acid	Pericarp	0.26 ± 0.01	0.29 ± 0.02	0.36 ±0.02	0.38 ± 0.01	0.40 ± 0.035
		Placenta	0.58 ± 0.04	0.75 ± 0.03	0.51 ± 0.01	0.76 ± 0.03	1.06 ± 0.09
	Fumaric acid	Pericarp	0.04 ± 0.01	0.04 ± 0.01	0.07 ± 0.01 **	0.06 ± 0.01	0.04 ± 0.01
		Placenta	0.25 ± 0.02	0.16 ± 0.01	0.16 ± 0.01	0.21 ± 0.02	0.13 ± 0.01
	Oxalic acid	Pericarp	1.49 ± 0.10	1.66 ± 0.15	2.74 ± 0.25	5.55 ± 0.29 ***	2.96 ± 0.23
		Placenta	5.23 ± 0.43	3.74 ± 0.32	5.41 ± 0.35	9.49 ± 0.72 ***	6.09 ± 0.59
	**Total organic acids**	**Pericarp**	**164.81 ± 10.46**	**149.61 ± 11.42**	**190.17 ± 12.52**	**226.51 ± 14.52**	**178.11 ± 13.24**
		**Placenta**	**165.12 ± 9.73**	**153.55 ± 10.52**	**163.82 ± 11.52**	**194.67 ± 14.42**	**227.02 ± 15.52**
*C. chinense* ‘Habanero Orange’	Ascorbic acid	Pericarp	12.95 ± 1.08	14.61 ± 0.94	15.68 ± 1.17	16.62 ± 1.29	
		Placenta	1.54 ± 0.04	2.27 ± 0.07	1.39 ± 0.06	2.27 ± 0.11	
	Citric acid	Pericarp	36.32 ± 3.52	35.73 ± 4.03	38.47 ± 2.12	44.22 ± 2.32	
		Placenta	41.82 ± 3.68	33.07 ± 3.42 **	23.92 ± 1.62 ***	36.62 ± 2.74	
	Malic acid	Pericarp	27.36 ± 2.56	27.12 ± 1.94	18.14 ± 1.37 **	16.06 ± 1.23 **	
		Placenta	44.37 ± 4.37	49.84 ± 4.23	32.73 ± 2.37	44.26 ± 4.63	
	Quinic acid	Pericarp	11.92 ± 0.92	11.86 ± 1.16	11.22 ± 0.72	12.04 ± 0.94	
		Placenta	16.73 ± 0.72	14.22 ± 1.84	8.47 ± 0.45 **	13.13 ± 1.06	
	Succinic acid	Pericarp	0.29 ± 0.01	0.38 ±0.02	0.40 ± 0.03	0.38 ± 0.02	
		Placenta	0.64 ± 0.02	0.59 ± 0.03	0.39 ± 0.01	0.64 ± 0.01	
	Fumaric acid	Pericarp	0.15 ± 0.01	0.12 ± 0.01	0.07 ± 0.01	0.18 ± 0.01	
		Placenta	0.37 ± 0.01	0.24 ± 0.01	0.28 ± 0.01	0.29 ± 0.01	
	Oxalic acid	Pericarp	2.34 ± 0.22	2.65 ± 0.21	2.36 ± 0.10	3.76 ± 0.32	
		Placenta	2.10 ± 0.20	2.03 ± 0.19	1.33 ± 0.03	2.06 ± 0.13	
	**Total organic acids**	**Pericarp**	**138.79 ± 8.52**	**139.67 ± 9.42**	**131.04 ± 9.32**	**140.94 ± 9.15**	
		**Placenta**	**174.22 ± 10.52**	**158.82 ± 10.99**	**102.29 ± 7.24**	**151.56 ± 8.27**	
*C. baccatum* ‘Bishop Crown’	Ascorbic acid	Pericarp	5.47 ± 0.07	5.65 ± 0.01	6.64 ± 0.03	7.09 ± 0.05	5.44 ± 0.03
		Placenta	1.56 ± 0.06	1.35 ± 0.07	2.96 ± 0.14	1.59 ± 0.02	2.31 ± 0.15
	Citric acid	Pericarp	53.23 ± 4.25	65.22 ± 5.99	64.27 ± 6.6	65.35 ± 2.95	70.02 ± 5.68
		Placenta	83.72 ± 7.22	70.12 ± 5.67	57.73 ± 3.54 ***	87.23 ± 7.96	98.22 ± 7.15
	Malic acid	Pericarp	8.77 ± 0.25	9.97 ± 0.66	11.54 ± 1.02	5.54 ± 0.44	5.73 ± 0.56
		Placenta	14.27 ± 1.33	16.83 ± 1.34	18.06 ± 1.72	11.88 ± 0.82	13.65 ± 1.02
	Quinic acid	Pericarp	7.83 ± 0.62	7.72 ± 0.34	7.71 ± 0.49	8.63 ± 0.62	8.96 ± 0.22
		Placenta	6.43 ± 0.47	8.46 ± 0.72	7.57 ± 1.07	6.49 ± 0.55	5.62 ± 0.42
	Succinic acid	Pericarp	0.60 ± 0.02	0.47 ± 0.03	0.42 ± 0.02	0.55 ±0.04	0.43 ± 0.02
		Placenta	0.82 ± 0.06	0.71 ± 0.04	0.95 ± 0.07	0.87 ± 0.08	0.69 ± 0.06
	Fumaric acid	Pericarp	0.07 ± 0.01	0.09 ± 0.01	0.07 ± 0.01	0.06 ± 0.01	0.06 ± 0.01
		Placenta	0.10 ± 0.01	0.11 ± 0.01	0.09 ± 0.01	0.11 ± 0.01	0.08 ± 0.01
	Oxalic acid	Pericarp	0.40 ± 0.03	0.33 ± 0.01	0.19 ± 0.01 ***	0.17 ± 0.01 ***	0.16 ± 0.01 ***
		Placenta	1.55 ± 0.04	1.55 ± 0.12	2.08 ± 0.18	1.44 ± 0.12	1.31 ± 0.07
	**Total organic acids**	**Pericarp**	**107.69 ± 6.62**	**120.29 ± 7.10**	**121.32 ± 9.12**	**121.58 ± 8.42**	**126.25 ± 9.42**
		**Placenta**	**134.26 ± 8.48**	**132.62 ± 9.42**	**119.14 ± 9.22**	**135.20 ± 10.49**	**144.22 ± 10.85**

* symbol indicates statistical significant differences of each fruit and fruit part when comparing to the first fruit on the plant. Significance codes: *** ≤ 0.001; ** ≤ 0.01; * ≤ 0.05; ‘empty slot’ ≥ 0.05.

**Table 4:** Total phenolic content (g GAE/kg DW, mean ± SE) in different species and three fruit parts.

Species	Fruit Part	Level 1	Level 2	Level 3	Level 4	Level 5
		Mean ± SE	Mean ± SE	Mean ± SE	Mean ± SE	Mean ± SE
*C. annuum* ‘Cayenne’	Pericarp	20.00 ± 0.67	22.26 ± 0.14	19.99 ± 0.16	21.59 ± 0.12	19.83 ± 0.11
	Placenta	27.85 ± 0.04	6.70 ± 0.03 ***	10.50 ± 0.08 ***	16.25 ± 0.10	14.69 ± 0.92 *
	Seed	2.66 ± 0.02	2.81 ± 0.01	2.68 ± 0.01	2.38 ± 0.02	2.57 ± 0.01
	**Whole fruit**	**10.76 ± 0.06**	7.75 ± 0.02 ***	7.82 ± 0.05 ***	8.96 ± 0.06 *	8.43 ± 0.04 **
*C. chinense* ‘Habanero Orange’	Pericarp	17.59 ± 0.06	20.25 ± 0.07	19.03 ± 0.04	19.70 ± 0.10	
	Placenta	30.43 ± 0.19	30.28 ± 0.12	31.46 ± 0.09	25.89 ± 0.17	
	Seed	3.86 ± 0.03	3.73 ± 0.03	4.09 ± 0.03	4.34 ± 0.04	
	**Whole fruit**	**12.19 ± 0.10**	**12.61 ± 0.11**	**12.82 ± 0.10**	**11.93 ± 0.09**	
*C. baccatum* ‘Bishop Crown’	Pericarp	10.02 ± 0.08	9.75 ± 0.04	10.31 ± 0.06	11.42 ± 0.06	10.82 ± 0.09
	Placenta	8.61 ± 0.04	19.01 ± 0.17 ***	13.47 ± 0.11 *	12.30 ± 0.12	12.12 ± 0.07
	Seed	2.08 ± 0.01	2.51 ± 0.01	1.87 ± 0.01	1.76 ± 0.01	1.69 ± 0.01
	**Whole fruit**	**5.01 ± 0.03**	**6.44 ± 0.03**	**5.54 ± 0.04**	**5.63 ± 0.03**	**5.40 ± 0.05**

* symbol indicates statistical significant differences of each fruit and fruit part when comparing to the first fruit on the plant. Significance codes: *** ≤ 0.001; ** ≤ 0.01; * ≤ 0.05; ‘empty slot’ ≥ 0.05.

**Table 5:** Individual and total capsaicinoids contents (g/kg DW, mean ± SE) in different species and three fruit parts.

Species	Capsaicinoid	Fruit Part	Fruit 1	Fruit 2	Fruit 3	Fruit 4	Fruit 5
			Mean ± SE	Mean ± SE	Mean ± SE	Mean ± SE	Mean ± SE
*C. annuum* ‘Cayenne’	Capsaicin	Pericarp	0.58 ± 0.03	1.15 ± 0.13	1.09 ± 0.12	0.92 ± 0.06	0.64 ± 0.13
		Placenta	9.67 ± 1.36	2.10 ± 0.32 ***	2.27 ± 0.63 ***	4.23 ± 0.56	2.83 ± 0.05 *
		Seeds	0.62 ± 0.04	0.56 ± 0.04	0.67 ± 0.05	0.41 ± 0.04	0.37 ± 0.05
	Dihydrocapsaicin	Pericarp	0.12 ± 0.67	0.20 ± 0.04	0.20 ± 0.01	0.15 ± 0.01	0.11 ± 0.02
		Placenta	2.70 ± 0.21	0.56 ± 0.02 ***	0.42 ± 0.07 ***	1.15 ± 0.07	1.04 ± 0.09 ***
		Seeds	0.10 ± 0.01	0.09 ± 0.05	0.01 ± 0.01	0.05 ± 0.01	0.04 ± 0.01
	Nordihydrocapsaicin	Pericarp	0.11 ± 0.21	0.22 ± 0.05	0.20 ± 0.01	0.17 ± 0.02	0.18 ± 0.05
		Placenta	2.03 ± 0.04	0.43 ± 0.03 ***	0.40 ± 0.07 ***	0.79 ± 0.03 *	0.53 ± 0.04 *
		Seeds	0.10 ± 0.01	0.11 ± 0.03	0.01 ± 0.01	0.07 ± 0.02	0.06 ± 0.02
	Homocapsaicin	Pericarp	0.03 ± 0.01	0.06 ± 0.03	0.06 ± 0.01	0.06 ± 0.01	0.05 ± 0.01
		Placenta	0.69 ± 0.11	0.17 ± 0.01 **	0.13 ± 0.02 ***	0.43 ± 0.06	0.34 ± 0.04
		Seeds	0.03 ± 0.01	0.03 ± 0.01	0.04 ± 0.01	0.03 ± 0.01	0.02 ± 0.01
	Homodihydrocapsaicin	Pericarp	0.04 ± 0.01	0.11 ± 0.05	0.10 ± 0.01	0.09 ± 0.01	0.08 ± 0.02
		Placenta	1.04 ± 0.26	0.30 ± 0.01	0.21 ± 0.02	0.63 ± 0.04	0.58 ± 0.03
		Seeds	0.05 ± 0.02	0.04 ± 0.01	0.06 ± 0.01	0.04 ± 0.01	0.03 ± 0.02
	**Total capsaicinoids**	**Pericarp**	**0.90 ± 0.32**	**1.76 ± 0.12**	**1.66 ± 0.15**	**1.41 ± 0.10**	**1.07 ± 0.25**
		**Placenta**	**16.15 ± 0.70**	3.57 ± 0.40 ***	3.45 ± 0.81 ***	**7.25 ± 0.52**	5.34 ± 0.16 *
		**Seeds**	**0.91 ± 0.07**	**0.84 ± 0.03**	**1.03 ± 0.08**	**0.62 ± 0.07**	**0.55 ± 0.09**
*C. chinense* ‘Habanero Orange’	Capsaicin	Pericarp	6.57 ± 0.59	6.51 ± 0.38	6.54 ± 0.25	6.31 ± 0.43	
		Placenta	17.52 ± 0.18	17.27 ± 0.28	12.48 ± 0.39	15.27 ± 0.18	
		Seeds	1.70 ± 0.13	1.63 ± 0.06	2.72 ± 0.05	1.97 ± 0.03	
	Dihydrocapsaicin	Pericarp	0.58 ± 0.03	0.66 ± 0.07	0.67 ± 0.03	0.73 ± 0.11	
		Placenta	3.17 ± 0.18	3.35 ± 0.48	1.96 ± 0.53	2.46 ± 0.47	
		Seeds	0.16 ± 0.04	0.17 ± 0.06	0.30 ± 0.07	0.22 ± 0.04	
	Nordihydrocapsaicin	Pericarp	0.22 ± 0.02	0.19 ± 0.06	0.28 ± 0.02	0.27 ± 0.02	
		Placenta	1.50 ± 0.11	1.70 ± 0.34	0.96 ± 0.28	1.23 ± 0.09	
		Seeds	0.08 ± 0.02	0.09 ± 0.03	0.01 ± 0.02	0.01 ± 0.02	
	Homocapsaicin	Pericarp	0.10 ± 0.01	0.11 ± 0.01	0.12 ± 0.01	0.12 ± 0.01	
		Placenta	0.53 ± 0.04	0.76 ± 0.11	0.34 ± 0.09	0.38 ± 0.01	
		Seeds	0.03 ± 0.01	0.04 ± 0.01	0.05 ± 0.01	0.03 ± 0.01	
	Homodihydrocapsaicin	Pericarp	0.08 ± 0.02	0.10 ± 0.01	0.12 ± 0.01	0.11 ± 0.01	
		Placenta	0.73 ± 0.04	0.82 ± 0.21	0.54 ± 0.0.1	0.77 ± 0.01	
		Seeds	0.02 ± 0.01	0.03 ± 0.01	0.06 ± 0.01	0.03 ± 0.01	
	**Total capsaicinoids**	**Pericarp**	**7.56 ± 0.65**	**7.59 ± 0.40**	**7.75 ± 0.21**	**7.56 ± 0.58**	
		**Placenta**	**23.47 ± 0.23**	**23.92 ± 0.39**	**16.29 ± 0.47**	**20.13 ± 0.16**	
		**Seeds**	**2.00 ± 0.43**	**1.98 ± 0.76**	**3.30 ± 0.70**	**2.37 ± 0.43**	
*C. baccatum* ‘Bishop Crown’	Capsaicin	Pericarp	0.44 ± 0.03	0.36 ± 0.05	0.47 ± 0.07	0.36 ± 0.11	0.23 ± 0.01
		Placenta	4.58 ± 0.11	9.97 ± 0.15 **	7.05 ± 0.47 *	6.84 ± 1.35	6.25 ± 0.41
		Seeds	0.38 ± 0.10	1.09 ± 0.48	0.37 ± 0.08	0.43 ± 0.10	0.30 ± 0.06
	Dihydrocapsaicin	Pericarp	0.08 ± 0.02	0.07 ± 0.01	0.09 ± 0.01	0.08 ± 0.01	0.04 ± 0.01
		Placenta	0.64 ± 0.01	1.41 ± 0.14	1.36 ± 0.21	1.23 ± 0.25	1.06 ± 0.15
		Seeds	0.06 ± 0.01	0.22 ± 0.01 ***	0.08 ± 0.01	0.09 ± 0.01	0.06 ± 0.01
	Nordihydrocapsaicin	Pericarp	0.02 ± 0.01	0.02 ± 0.01	0.03 ± 0.01	0.03 ± 0.01	0.01 ± 0.01
		Placenta	0.30 ± 0.05	0.80 ± 0.08	0.65 ± 0.01	0.64 ± 0.11	0.62 ± 0.07
		Seeds	0.01 ± 0.00	0.04 ± 0.01 ***	0.02 ± 0.00	0.01 ± 0.00	0.01 ± 0.00
	Homocapsaicin	Pericarp	0.01 ± 0.01	0.01 ± 0.01	0.02 ± 0.01 **	0.01 ± 0.01 **	0.01 ± 0.00
		Placenta	0.16 ± 0.04	0.32 ± 0.07	0.31 ± 0.06	0.38 ± 0.14	0.29 ± 0.03
		Seeds	0.01 ± 0.01	0.01 ± 0.00 **	0.01 ± 0.00	0.01 ± 0.00 **	0.01 ± 0.00
	Homodihydrocapsaicin	Pericarp	0.01 ± 0.00	0.01 ± 0.00	0.01 ± 0.00	0.01 ± 0.00	0.01 ± 0.00
		Placenta	0.07 ± 0.01	0.27 ± 0.05 ***	0.17 ± 0.02	0.19 ± 0.05	0.18 ± 0.03
		Seeds	0.01 ± 0.00	0.01 ± 0.00 ***	0.01 ± 0.00	0.01 ± 0.00	0.01 ± 0.00
	**Total capsaicinoids**	**Pericarp**	**58.33 ± 5.90**	**48.66 ± 8.07**	**61.98 ± 11.12**	**50.35 ± 16.27**	**30.90 ± 1.56**
		**Placenta**	**5.78 ± 0.13**	**12.77 ± 1.82**	**9.56 ± 0.78**	**9.30 ± 0.19**	**8.42 ± 0.70**
		**Seeds**	**0.48 ± 0.12**	**1.40 ± 0.61**	**0.49 ± 0.10**	**0.56 ± 0.13**	**0.40 ± 0.10**

* symbol indicates statistical significant differences of each fruit and fruit part when comparing to the first fruit on the plant. Significance codes: *** ≤ 0.001; ** ≤ 0.01; * ≤ 0.05; ‘empty slot’ ≥ 0.05.

## Data Availability

The data presented in this study are available on request from the corresponding author. The data are not publicly available due to privacy.

## Supplementary Materials

The following are available online at https://www.mdpi.com/article/10.3390/plants11010101/s1, Figure S1: Chromatogram of sugars in chillies. Peak A: Sucrose; Peak B: Glucose; Peak C: Fructose, Figure S2: Chromatogram of organic acids (Figure S2A,B) in chillies. Figure S2A: 1 = Oxalic acid; 2 = Citric acid; 3 = Malic acid; 4 = Quinic acid; 5 = Succinic acid; 6 = Fumaric acid; Figure S2B: 1 = Ascorbic acid, Figure S3: Cromatograph of capsaicin standard detected with quadropole MS., Figure S4: Cromatograph of dihydrocapsaicin standard detected with quadropole MS., Figure S5: Cromatograph of nordihydrocapsaicin standard detected with quadropole MS.

## References

[1] Welbaum G. (2015). Vegetable Production and Practices.

[2] Fett D.D. (2003). Botanical briefs: *Capsicum* peppers. Cutis.

[3] Al Othman Z.A., Ahmed Y.B., Habila M.A., Ghafar A.A. (2011). Determination of capsaicin and dihydrocapsaicin in *Capsicum* fruit samples using high performance liquid chromatography. Molecules.

[4] Zewdie Y., Bosland P. (2000). Pungency of Chile (*Capsicum annuum* L.) Fruit is Affected by Node Position. HortScience.

[5] Erickson A., Markhart A.H. (2002). Flower developmental stage and organ sensitivity of bell pepper (*Capsicum annuum* L.) to elevated temperature. Plant Cell Environ..

[6] Orobiyi A. (2015). Capsaicin and Ascorbic Acid Content in the High Yielding Chili Pepper (*Capsicum annuum* L.) Landraces of Northern Benin. Int. J. Curr. Microbiol. Appl. Sci..

[7] Verma N., Shukla S. (2015). Impact of various factors responsible for fluctuation in plant secondary metabolites. J. Appl. Res. Med. Aromat. Plants.

[8] Yang L., Wen K.S., Ruan X., Zhao Y.X., Wei F., Wang Q. (2018). Response of Plant Secondary Metabolites to Environmental Factors. Molecules.

[9] Fayos O., Ochoa-Alejo N., de la Vega O.M., Savirón M., Orduna J., Mallor C., Barbero G.F., Garcés-Claver A. (2019). Assessment of Capsaicinoid and Capsinoid Accumulation Patterns during Fruit Development in Three Chili Pepper Genotypes (*Capsicum* spp.) Carrying Pun1 and pAMT Alleles Related to Pungency. J. Agric. Food Chem..

[10] De Ávila Silva L., Condori-Apfata J.A., Marcelino M.M., Tavares A.C.A., Raimundi S.C.J., Martino P.B., Araújo W.L., Zsögön A., Sulpice R., Nunes-Nesi A. (2019). Nitrogen differentially modulates photosynthesis, carbon allocation and yield related traits in two contrasting *Capsicum chinense* cultivars. Plant Sci. Int. J. Exp. Plant Biol..

[11] Zamljen T., Veberic R., Hudina M., Slatnar A. (2021). The Brown Marmorated Stink Bug (*Halyomorpha halys* Stål.) Influences Pungent and Non-Pungent *Capsicum* Cultivars’ Pre- and Post-Harvest Quality. Agronomy.

[12] Zamljen T., Zupanc V., Slatnar A. (2020). Influence of irrigation on yield and primary and secondary metabolites in two chilies species, *Capsicum annuum* L. and *Capsicum chinense* Jacq. Agric. Water Manag..

[13] Zamljen T., Hudina M., Veberič R., Slatnar A. (2021). Biostimulative effect of amino acids and green algae extract on capsaicinoid and other metabolite contents in fruits of *Capsicum* spp.. Chem. Biol. Technol. Agric..

[14] Zamljen T., Jakopič J., Hudina M., Veberič R., Slatnar A. (2021). Influence of intra and inter species variation in chilies (*Capsicum* spp.) on metabolite composition of three fruit segments. Sci. Rep..

[15] Medic A., Jakopic J., Hudina M., Solar A., Veberic R. (2021). Identification and quantification of the major phenolic constituents in Juglans regia L. peeled kernels and pellicles, using HPLC–MS/MS. Food Chem..

[16] R Core Team (2021). R: A Language and Environment for Statistical Computing.

[17] Alan Ö., Eser B. (2008). Pepper seed yield and quality in relation to fruit position on the mother plant. PJBS.

[18] Krstić B., Tepić Horecki A., Nikolić N., Dj G., Tomicic M. (2013). Chemical variability of inedible fruit parts in pepper varieties (*Capsicum annuum* L.). Bulg. J. Agric. Sci..

[19] Pandhair V., Sharma S. (2008). Accumulation of Capsaicin in Seed, Pericarp and Placenta of *Capsicum annuum* L Fruit. J. Plant Biochem. Biotechnol..

[20] Aldana-Iuit J.G., Sauri-Duch E., Miranda-Ham M.D.L., Castro-Concha L.A., Cuevas-Glory L.F., Vázquez-Flota F.A. (2015). Nitrate Promotes Capsaicin Accumulation in *Capsicum chinense* Immobilized Placentas. BioMed Res. Int..

[21] Gavilán Guillen N., Tito R., Gamarra Mendoza N. (2018). Capsaicinoids and pungency in *Capsicum chinense* and *Capsicum baccatum* fruits1. Pesqui. Agropecu. Trop..

[22] Loizzo M.R., Pugliese A., Bonesi M., De Luca D., O’Brien N., Menichini F., Tundis R. (2013). Influence of drying and cooking process on the phytochemical content, antioxidant and hypoglycaemic properties of two bell *Capsicum annum* L. cultivars. Food Chem. Toxicol..

[23] Loizzo M.R., Pugliese A., Bonesi M., Menichini F., Tundis R. (2015). Evaluation of chemical profile and antioxidant activity of twenty cultivars from *Capsicum annuum*, *Capsicum baccatum*, *Capsicum chacoense* and *Capsicum chinense*: A comparison between fresh and processed peppers. LWT.

[24] Jarret R., Berke T., Baldwin E., Antonious G. (2009). Variability for Free Sugars and Organic Acids in *Capsicum chinense*. Chem. Biodivers..

[25] Iwai K., Suzuki T., Fujiwake H.J.A. (1979). Formation and Accumulation of Pungent Principle of Hot Pepper Fruits, Capsaicin and Its Analogues, in *Capsicum annuun* var. annuun cv. Karayatsubusa at Different Growth Stages after Flowering. Agric. Biol. Chem..

[26] Lewallen K., Marini R. (2003). Relationship between Flesh Firmness and Ground Color in Peach as Influenced by Light and Canopy Position. J. Am. Soc. Hortic. Sci..

[27] Willaume M., Lauri P.-E., Sinoquet H. (2004). Light interception in apple trees influenced by canopy architecture manipulation. Trees.

[28] Ghasemnezhad M., Sherafati M., Payvast G.A. (2011). Variation in phenolic compounds, ascorbic acid and antioxidant activity of five coloured bell pepper (*Capsicum annum*) fruits at two different harvest times. J. Funct. Foods.

[29] Contreras-Padilla M., Yahia E.M. (1998). Changes in Capsaicinoids during Development, Maturation, and Senescence of Chile Peppers and Relation with Peroxidase Activity. J. Agric. Food Chem..

[30] Estrada B., Bernal M.A., Díaz J., Pomar F., Merino F. (2002). Capsaicinoids in vegetative organs of *Capsicum annuum* L. in relation to fruiting. J. Agric. Food Chem..

[31] Coyago-Cruz E., Corell M., Moriana A., Hernanz D., Stinco C.M., Meléndez-Martínez A.J. (2017). Effect of the fruit position on the cluster on fruit quality, carotenoids, phenolics and sugars in cherry tomatoes (*Solanum lycopersicum* L.). Food Res. Int..

[32] Kalcsits L., Mattheis J., Giordani L., Reid M., Mullin K., Beres B. (2019). Fruit canopy positioning affects fruit calcium and potassium concentrations, disorder incidence, and fruit quality for ‘Honeycrisp’ apple. Can. J. Plant Sci..

[33] Feng F., Li M., Ma F., Cheng L. (2014). Effects of location within the tree canopy on carbohydrates, organic acids, amino acids and phenolic compounds in the fruit peel and flesh from three apple (*Malus* × *domestica*) cultivars. Hortic. Res..

[34] Tundis R., Loizzo M.R., Menichini F., Bonesi M., Conforti F., De Luca D., Menichini F. (2012). Air-dried *Capsicum annuum* var. acuminatum medium and big: Determination of bioactive constituents, antioxidant activity and carbohydrate-hydrolyzing enzymes inhibition. Food Res. Int..

