# Supplementary material for: Metabolic Variation among Fruits of Different Chili Cultivars (Capsicum spp.) Using HPLC/MS
Source: Plants (Basel). 2021 Dec 29;11(1):101. doi: 10.3390/plants11010101 (PMC8747607; doi:10.3390/plants11010101)
Supplement: Supplementary file 1 [file plants-11-00101-s001.zip › plants-1537001-supplementary.pdf]

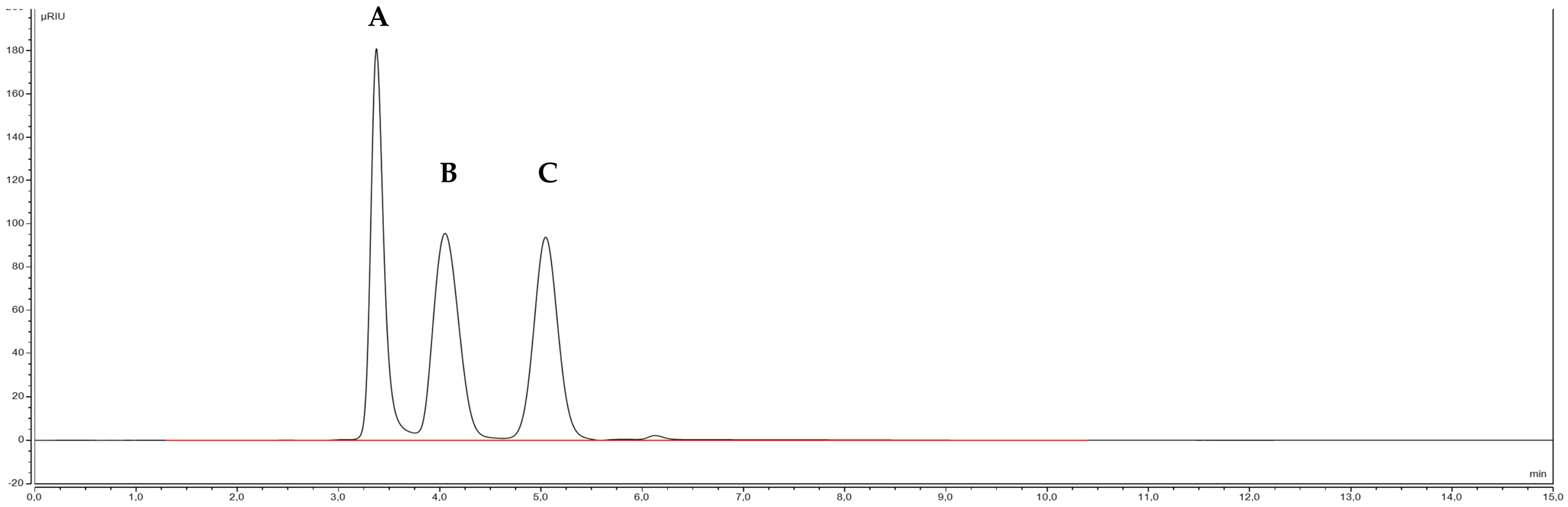

Figure S1: Chromatogram of sugars in chillies. **Peak A**: Sucrose**; Peak B**: Glucose; **Peak C**: Fructose.

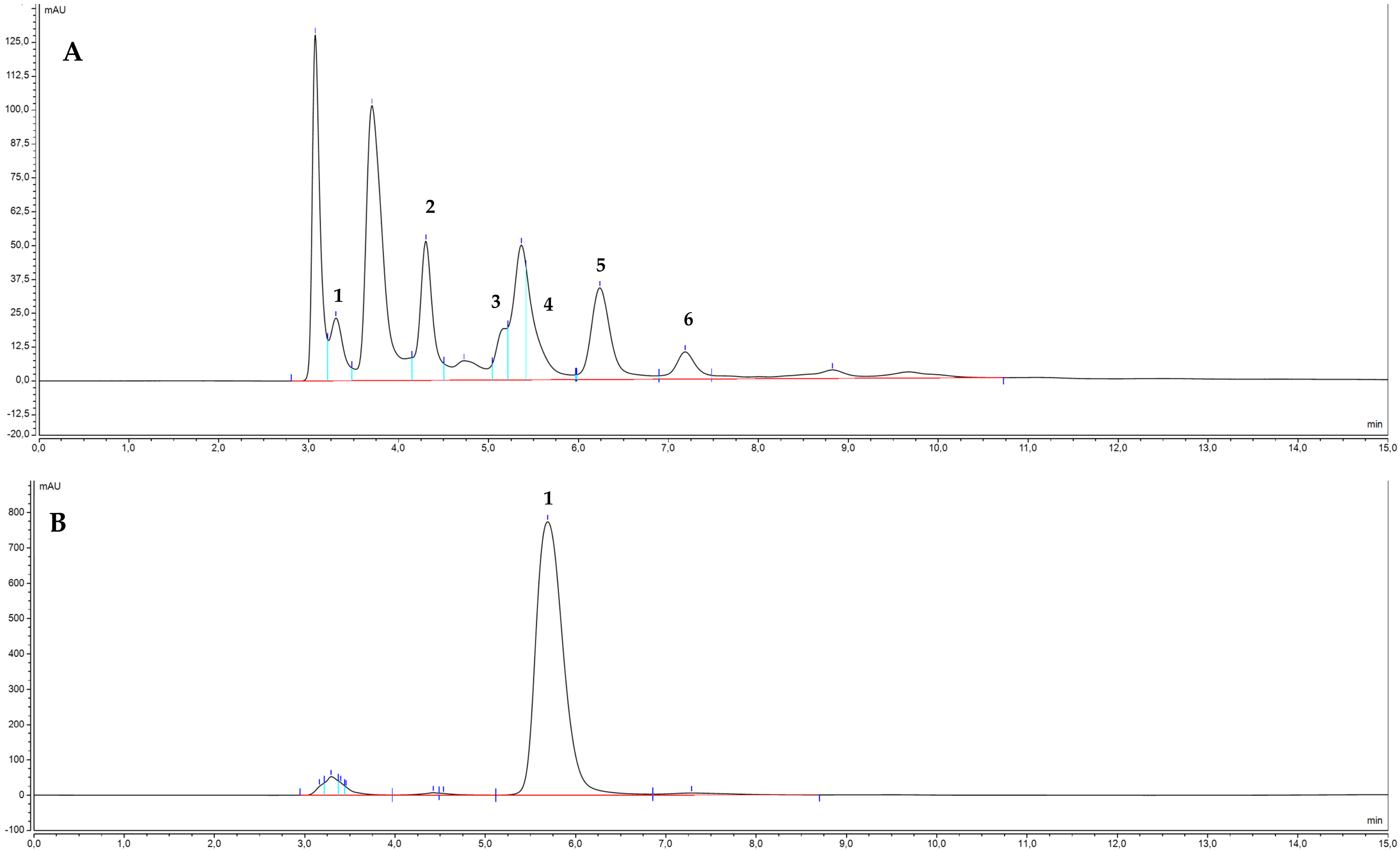

Figure S2: Chromatogram of organic acids (Figure A and B) in chillies. Figure A: 1 = Oxalic acid; 2 = Citric acid; 3 = Malic acid; 4 = Quinic acid; 5 = Succinic acid; 6 = Fumaric acid; Figure B: 1 = Ascorbic acid

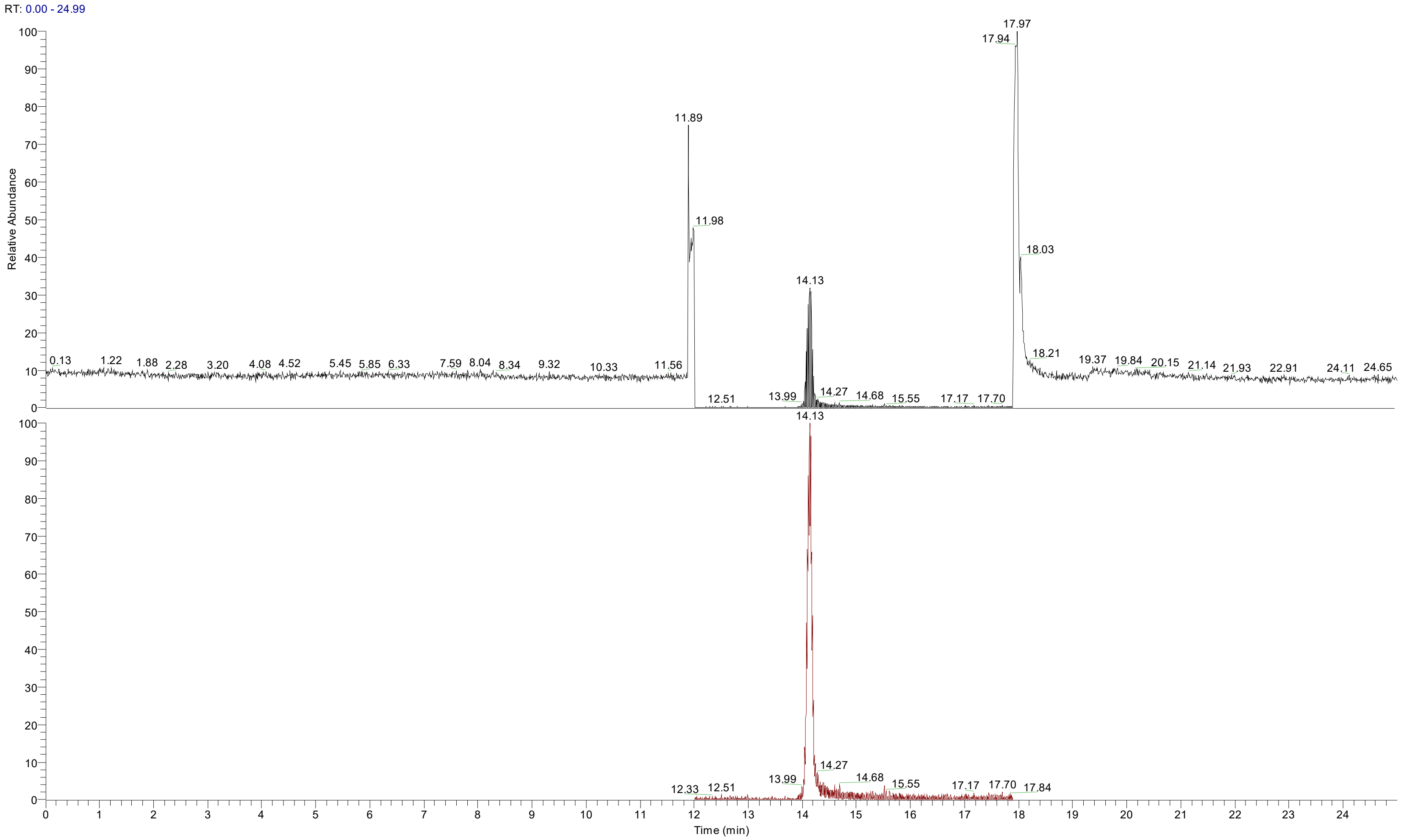

Figure S3: Cromatograph of capsaicin standard detected with quadropole MS.

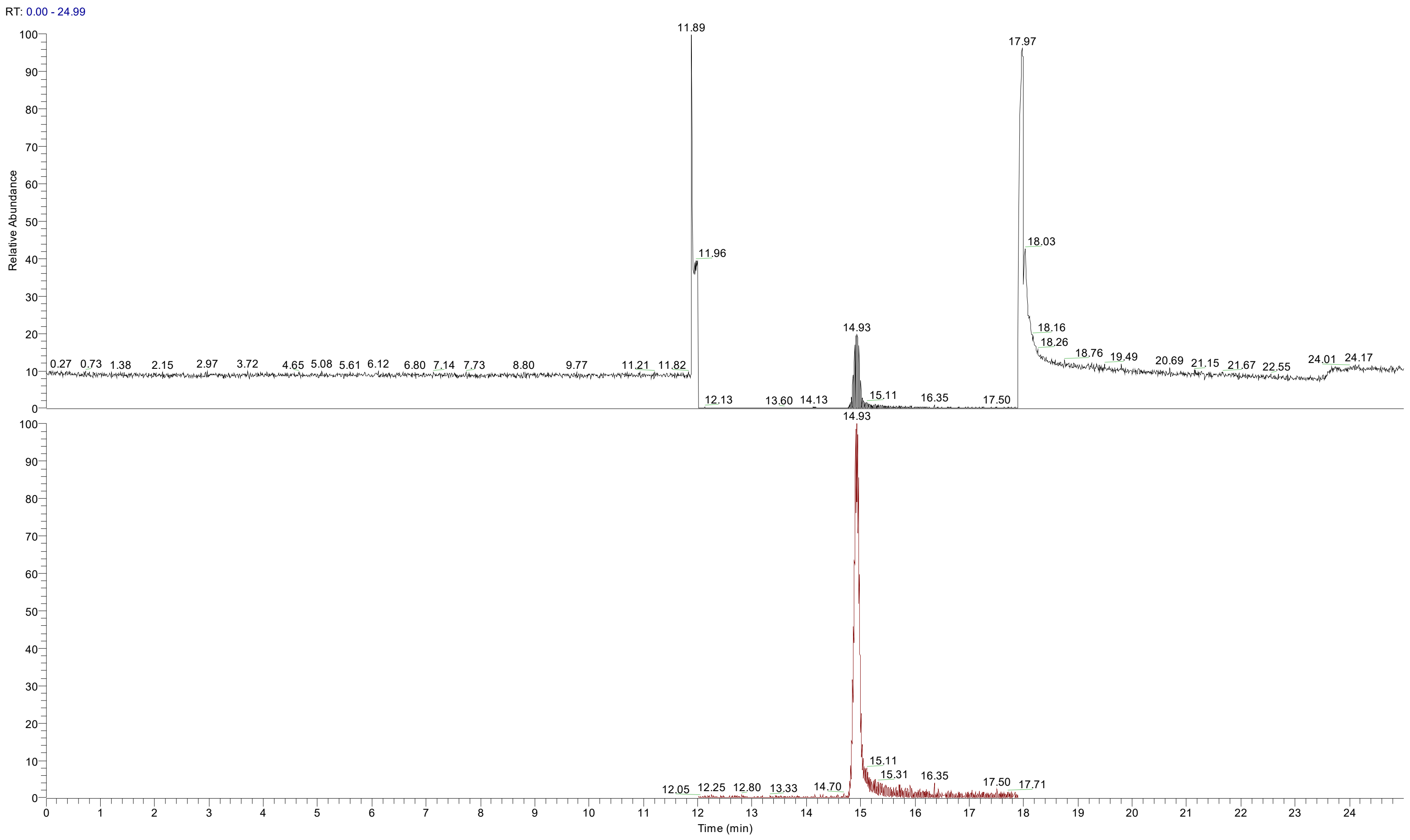

Figure S4: Cromatograph of dihydrocapsaicin standard detected with quadropole MS.

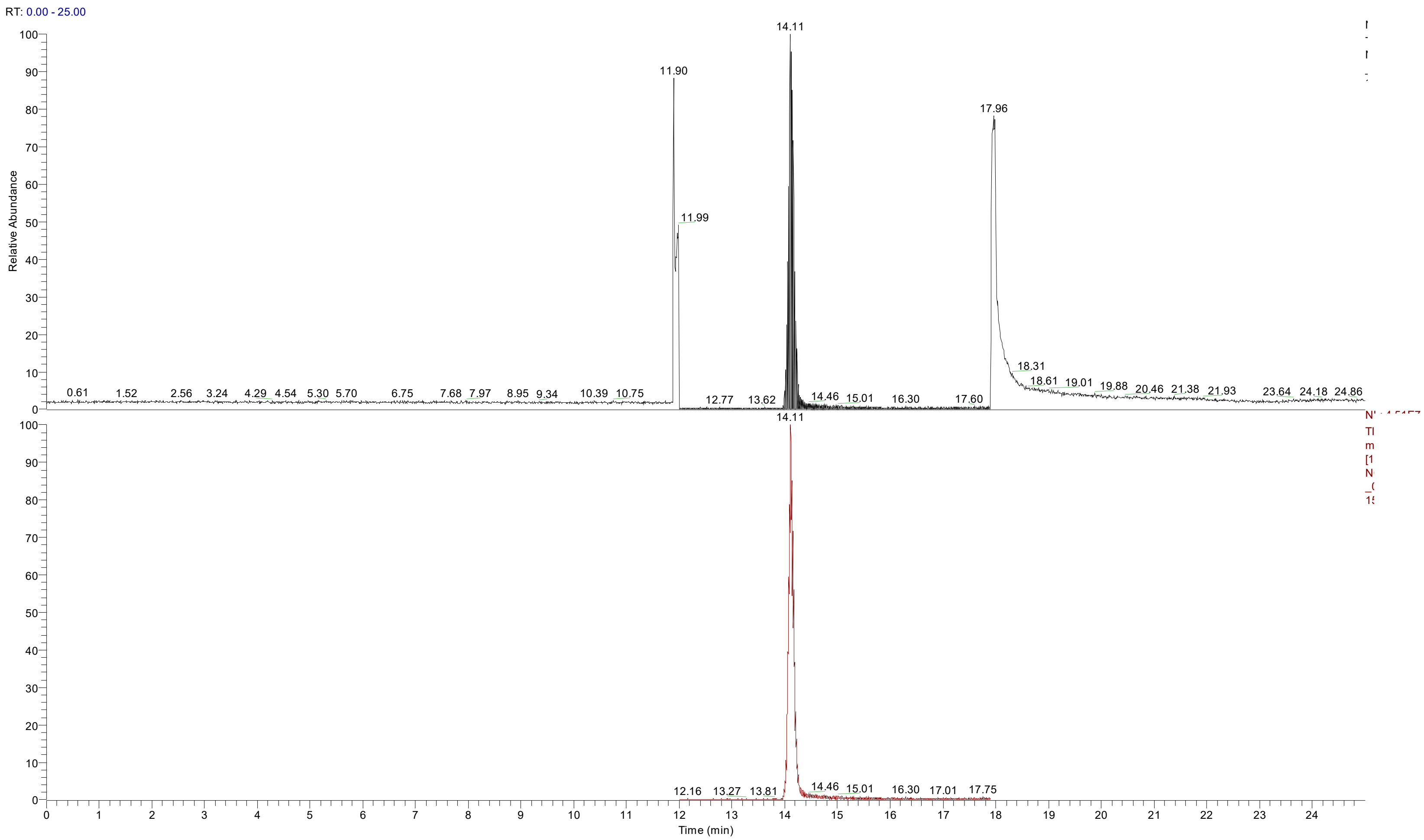

Figure S5: Cromatograph of nordihydrocapsaicin standard detected with quadropole MS
